# Plasmonic ultraviolet filter for fast-timing applications

**DOI:** 10.1515/nanoph-2022-0704

**Published:** 2023-01-23

**Authors:** Ryosuke Ota, Soh Uenoyama

**Affiliations:** Central Research Laboratory, Hamamatsu Photonics K.K., 5000 Hirakuchi, Hamakita-ku, Hamamatsu City 434-8601, Japan

**Keywords:** barium fluoride, plasmonic filter, surface plasmon resonance, time resolution

## Abstract

Barium fluoride, an inorganic scintillation material used for the detection of X-ray and/or gamma-ray radiation, has been receiving increasing attention in the field of radiation measurements in fast-timing applications. To make full use of its timing properties, its slow emission around the ultraviolet region, more specifically, the 300 nm region needs to be suppressed. Although doping ions, such as lanthanum, yttrium, and cadmium, can suppress the slow component, such techniques can lose information of interacted radiations. Consequently, a suppression technique that does not suffer from information loss while maintaining precise timing measurements would be desirable. In this study, we proposed aluminum nano-disk-based plasmonic filters to suppress slow emissions while maintaining fast emissions around 195 and 220 nm and a usability of the slow component. Finite-difference time-domain simulations and experimental results exhibited good agreement, with over 90% of slow components being adequately suppressed without sacrificing fast components, proving that aluminum nanodisks can be used for ultraviolet filters. Moreover, based on the designed filter performance, we conducted coincidence time resolution simulations for positron–electron annihilation gamma rays from an analytical perspective. The simulations indicated the designed filters could maintain high timing performance. Consequently, the proposed plasmonic ultraviolet filter was suitable for maximizing the potential of barium fluoride scintillators.

## 1 Introduction

Radiation measurement with high temporal precision—that is, of the order of tens of picoseconds (ps) or better—has been a requirement in various applications, including high-energy physics experiments, and nuclear medicine including time-of-flight positron emission tomography (TOF-PET) [1–6]. In physics experiments, particle identification, particle trajectory reconstruction, and pile-up mitigation can benefit from fast-timing performance, while in TOF-PET the enhanced signal-to-noise ratio of PET images and reconstruction-free imaging can benefit from it [7, 8]. Scintillators coupled to photodetectors—such as the photomultiplier tube (PMT) and silicon photomultiplier (SiPM) [9] are often used for such applications, with lutetium oxyorthosilicate (LSO) and lutetium–yttrium oxyorthosilicate (LYSO) having been considered as fast scintillators. Given their scintillation properties, a coincidence time resolution (CTR) better than 100 ps in full width at half maximum (FWHM) for 511 keV positron-electron annihilation gamma rays can be theoretically and experimentally achieved thanks to the development of scintillators, photodetectors, and estimation algorithms [10–15]. However, lutetium-based scintillators have the disadvantage of intrinsic radioisotopes and high production costs.

Recently, scintillators with cross-luminescence have once again garnered attention in fast-timing applications [16–19] owing to their ultrafast emission, although their mechanisms were investigated in the 1990s [20]. Barium fluoride (BaF_2_)—the production cost of which is only one fourth of that of LSO [21]—is a representative scintillator with cross-luminescence and was developed in the 1970s [22]. Generally, it has been reported that BaF_2_ has two decay components—that is, fast (*τ*_fast_ ∼0.6 ns at 195 and 220 nm) and slow (*τ*_slow_ ∼ 620 ns at 300 nm) decay time constants with light yields of 1400 and 9500 photons/MeV, respectively [23]. The light yield of 1400 photons/MeV with *τ*_fast_ ∼ 0.6 ns is denser in time than that of LSO/LYSO, thus the achievable intrinsic CTR of BaF_2_ should be better than that of LSO/LYSO. In fact, the CTR of BaF_2_ coupled to vacuum ultraviolet (VUV) SiPM achieved 51 ps FWHM despite the relatively low photodetection efficiency (PDE) of the SiPM in the VUV region (22% at 200 nm) [24, 25]. However, the slower decay component (*τ*_slow_ ∼ 620 ns at 300 nm) can cause baseline fluctuations which degrade the timing performance. Consequently, careful offline analysis or suppression techniques of the slow decay component are required to correctly pick off timing information from the photodetector signals. Although doping ions—such as lanthanum, yttrium, and cadmium—can suppress the slow component and were experimentally validated [26–28], this suppression means that they lost a lot of information (9500 photons/MeV), which can be used as energy information of interacted radiations. This indicates that the energy resolution would get worse by ion doping. Therefore, an alternative way to keep baseline stability without compromising energy information would be preferrable.

Nanophotonics technology has been applied to scintillators and photodetectors for radiation measurements [29–31]. In 1998, Ebessen et al. demonstrated the phenomena of extraordinary optical transmittance (EOT) using metallic periodic nano-hole arrays with a length thicker than that of the skin depth of the metal [32, 33]. This phenomenon was based on surface plasmon polaritons (SPPs), collective free electron oscillations at the interface between metal and dielectric which are excited when the period and shape of the nanostructure satisfies the resonance wavelength. Adjusting the shape and size of the nano-hole accordingly enables to precisely control of the transmission spectra, opening up attractive filtering applications including color and dynamic filters [34–36]—making such SPP-based filtering techniques highly appropriate for addressing the abovementioned requirements. However, an EOT-based nano-hole array does not exhibit high transmittance since it exhibits strong absorption at resonance wavelengths as well [37, 38].

Metallic nano-disk arrays with configurations complementary to metallic nano-hole array demonstrates extraordinarily low transmittance (ELT) characteristics despite the thickness of the metallic layer being comparable to the skin depth (or less) at resonance wavelengths [39]. Several research groups have demonstrated the ELT phenomena from visible to near infrared wavelengths using gold or silver nano-disk arrays [40, 41]. However, free electrons within gold or silver can be difficult to follow in the (near) UV range—specifically 300 nm for the BaF_2_ case. Consequently, a material for which the plasma frequency can follow the speed of (near) UV photons should be employed, and aluminum is the best material. Aluminum has many advantages in terms of the CMOS process, its cost, and its chemical stability against oxidation and sulfidation. Although aluminum nano-disk arrays have already been demonstrated at visible light wavelengths, they have not yet been demonstrated at (near) UV wavelengths [42].

**Figure 1:**
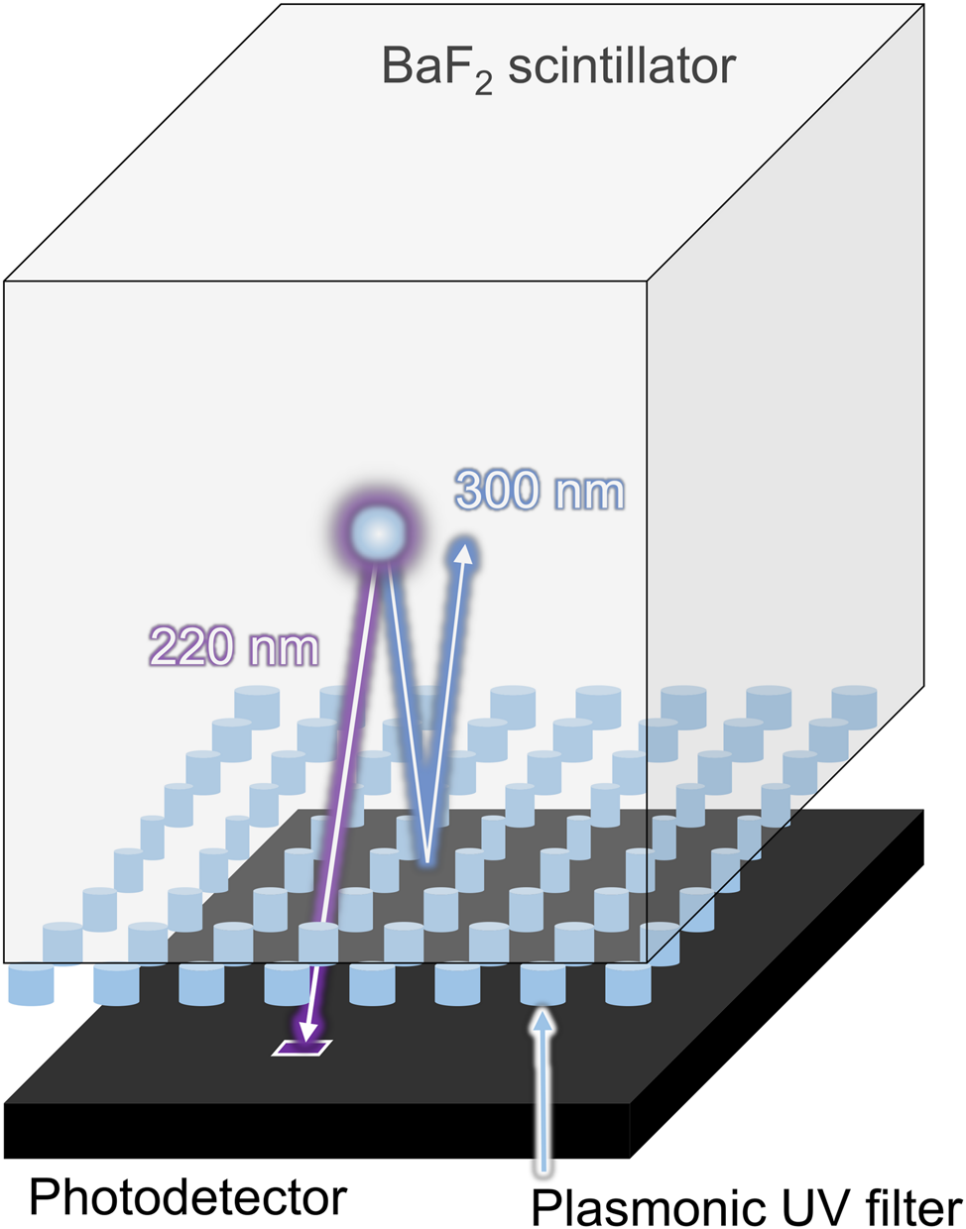
Conceptual schematic of the proposed UV filter based on the localized surface plasmon resonance applied to a BaF_2_ scintillator which can be used for fast timing applications. The plasmonic UV filter is transparent to the fast component (220 nm), while it can reflect the slow component (300 nm).

In this study, we proposed an aluminum-based plasmonic UV filter for a BaF_2_ scintillator as shown in Figure 1. We carefully designed the size and period of the nano disk to control the resonance wavelength of the localized surface plasmon resonance combined with SPP to obtain a high transmittance in the faster component (*λ* = 220 nm) and to simultaneously suppress the slow component (*λ* = 300 nm) while maintaining the usability of the suppressed slow component. (*λ* = 300 nm) while maintaining the usability of the suppressed slow component. In addition to finite-difference time-domain (FDTD) simulations, plasmonic UV filters were fabricated on a glass substrate and their transmissions were experimentally examined. Furthermore, analytical CTR simulations were also performed based on the filtering performance.

## 2 Results and discussion

**Figure 2:**
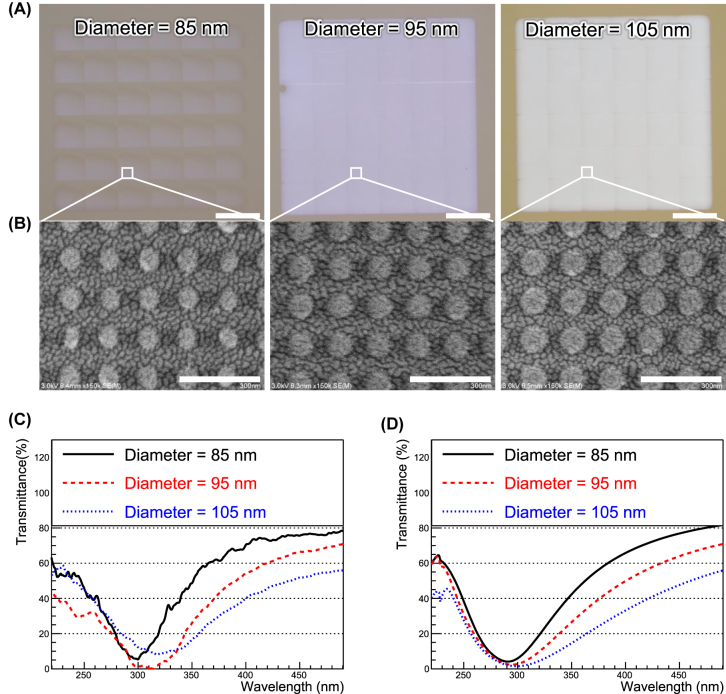
Microscopic images of fabricated plasmonic filters, and their experimental and simulations results. (A) Microscopic images of aluminum nanodisks of different diameters (85, 95, and 105 nm). Scale bar is 200 μm. (B) SEM images of aluminum nanodisks of different diameters (85, 95, and 105 nm). Scale bar is 300 nm. (C) and (D) Transmission spectra of aluminum nanodisks of different diameters (85, 95, and 105 nm) for experiments and simulations, respectively.

Figure 2(A) shows a microscopic image of the fabricated aluminum nano-disk arrays on a glass substrate (*ϕ* of 21.8 mm, *t* of 3 mm) using electric beam lithography (see Supplementary Material). The size of the nano-disk array is 900 × 900 μm^2^, each square comprising different diameters—that is, 85, 95, and 105 nm, respectively (left to right). Figure 2(B) shows scanning electron microscopy (SEM) images of the same. As is seen from Figure 2(B), the fabricated aluminum nano-disk array is fabricated as designed.

Figure 2(C) and (D) plots the transmittance of the aluminum nano-disk array for experiments and simulations, respectively, the experimental results demonstrating good agreement with the simulation results. The experimental results are obtained by using a spectrophotometer (UH4150, Hitahi High-Technologies Corporation), and the measurements were performed at wavelengths of 200–500 nm with 0.5 nm steps. Details can be found in Supplementary Material. In particular, the 90 nm diameter transmission spectrum is well-suited for suppressing the slow component of BaF_2_ emissions. The slight difference between the simulation and experimental results is because the actual refractive index differs from that used in the simulation as well as a few fabrication errors. The absolute number of experimental transmittances is normalized so that the transmittances of experiments around 500 nm correspond to those of the simulations. This was done because of the non-uniform surface quality of the glass substrate on which the filters were fabricated, making it difficult to measure the absolute number of transmittances. However, the fact remains that clear dips around *λ* = 300 nm are observed.

**Figure 3: j_nanoph-2022-0704_fig_003:**
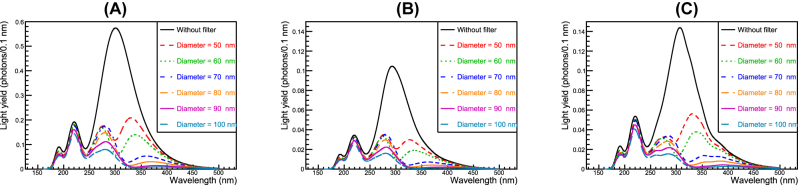
Emission spectra after being filtered using the proposed plasmonic filter: (A) ideal photodetector with perfect QE (100%), (B) multi-alkali photocathode, and (C) VUV SiPM. Choosing an optimal diameter of nanodisk enables suppression of slow components around 300 nm.

**Figure 4:**
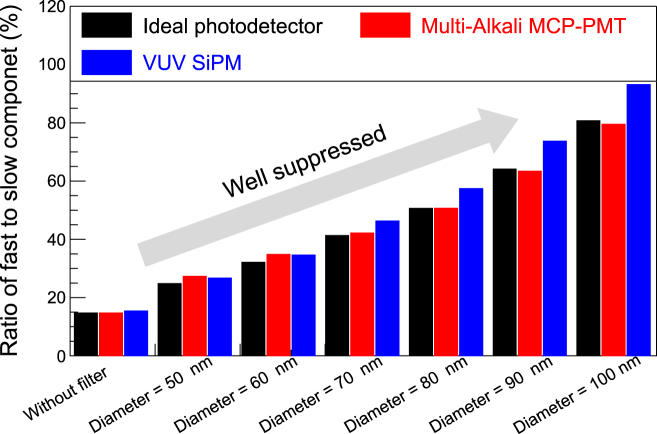
The ratio of fast to slow components of BaF_2_ after filtering. In the case of a diameter = 100 nm, the ratio more than 80–90% indicating that the designed filter can electrically stabilize the baseline of photodetectors.

After passing through the simulated filters and being detected by the modeled photodetectors (details of which are described in the Materials and Methods section), the emission spectra are shown in Figure 3—that is, (A) the ideal photodetector, (B) multi-alkali photocathode microchannel plate-PMT (MCP-PMT), and (C) VUV SiPM. The light yields (*Y-*axis) are clearly affected by the photodetectors’ absolute number of quantum efficiency (QE)/PDE. However, the most importance feature of Figure 3 is that the slow emission around 300 nm is greatly suppressed by the correct choice of filter parameter—that is, the diameter of the nanodisks. In the 100 nm diameter case, the emission spectra around 300 and 350 nm is less than 10% and 1%, respectively, compared to the without-filter case, while maintaining fast emission around 195/220 nm. The ratio of fast to slow emission is shown in Figure 4. As the diameter increases, the ratio increases to >90% for the VUV SiPM, which exhibits the best performance because it has a higher PDE at 220 nm than 300 nm while the QE of the multi-alkali device has a flatter curve.

**Figure 5:**
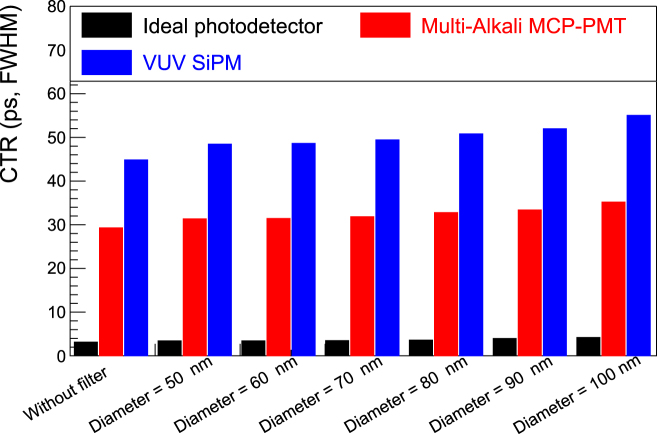
Analytically simulated CTRs with and without the designed filters. Irrespective of the diameter, the CTR does not change. This means the designed filters can maintain the BaF_2_ timing properties.

The analytically simulated CTRs are shown in Figure 5, which is the first approximation and takes photodetector models and scintillation kinetics into account but excludes the transit travel time of scintillation photons in a crystal. More quantitative information is summarized in Supplementary Material. In the case of the ideal photodetector, the CTRs are better than 10 ps FWHM for all filters, thanks to the high emission density of BaF_2_. However, in practice, given the finite dimensions of BaF_2_ crystals, the time spread of photons’ traveling in the crystal should be greater than 10 ps FWHM even if its thickness is only a few mm. Consequently, the ideal results suggest the intrinsic CTR. Conversely, the CTRs for the modeled MCP-PMT and VUV SiPM range from 30 to 35 ps and from 45 to 55 ps FWHM, respectively, still as good a CTR as the state-of-the-art detectors [25, 43].

The plasmonic ultraviolet filters were designed and experimentally developed, and their feasibility for use with BaF_2_ scintillators was investigated. Aluminum nanodisks of 30-nm thickness were fabricated. However, a thinner film thickness exhibits a higher transmittance in the FDTD simulations (see Supplementary Material). In future studies, we aim to achieve even higher transmittances by means of etching after the fabrication of the aluminum nanodisk arrays to reduce the film thickness.

The reason the simulation results (see Materials and Methods) have a resonance peak at shorter wavelengths than the experimental results is because the refractive index of the surrounding medium changes from optical grease (with a refractive index of 1.45)—which would be used in practical situations—to air (with a refractive index of 1) to be consistent with the experimental environment. Consequently, the nanodisk should be fabricated slightly smaller than its current size to connect them to the scintillator after optical grease has been applied.

Figures 2 [Fig j_nanoph-2022-0704_fig_003]–4 show the designed filter can suppress the slow component peaking around 300 nm, resulting in a considerable increase in the ratio of the fast to slow component without compromising the absolute number of fast photons. Here, the ratio of the fast to slow component is defined as follows.

(1) $$Ratio\equiv \frac{\begin{array}{c} Expected\;\;number\;\;of\;\;detected\;\;photons\\ for\;\;fast\;\;component\;\;\left(195\;\;and\;\;220\;\;nm\right) \end{array}}{\begin{array}{c} Expected\;\;number\;\;of\;\;detected\;\;photons\;\;for\\ slow\;\;component\;\;\left(300\;\;nm\right) \end{array}}$$

Therefore, the ratio is 14.7% for the ideal photodetector without filters (0.147 = 1400/9500 as listed in Table 1); then, it accordingly increases by applying the filters. Consequently, the baseline will be stable, making it easy to extract timing information from the photodetector signal. A filter of larger diameter further would suppress the slow component although in this study we only evaluated the diameter up to 100 nm. We could control and optimize the dip width by engineering both the period and diameter of the nanodisks. Consequently, an optimized filter design could maintain the number of fast photons while further discriminating against the slow photons. As the number of fast photons are maintained, there is sufficient room for a CTR < 100 ps FWHM using existing photodetectors. The MCP-PMT shows better CTR than the VUV SiPM despite the VUV SiPM having better PDE than the multi-alkali photocathode MCP-PMT, indicating that the CTR benefits more from single photon time resolution (SPTR) rather than QE/PDE because of the high emission density of BaF_2_ in time.

**Table 1:** Parameters of BaF_2_ emission used in the CTR simulation.

	Light yield (photons/MeV)	Decay time (ns)	Rise time (ns)	Emission wavelength (nm)
Fast	1400	0.6	0.001	192/220
Slow	9500	620	0.001	300

As shown in Figure 4, the ratio increases substantially while maintaining the fast component. Figure 5 shows that no explicit degradation in the CTR is evident for all filters. Although the fast component is sufficiently maintained, and a stabilized baseline can be expected, the photon density in time (which plays an important role for the timing performance) in the fast component slightly decreased, resulting in slight degradation in the CTR. However, the expected CTR is still maintained as that of the state-of-the-art detectors. Consequently, the high timing capability of BaF_2_ is sustained when the designed filters are used.

Compared to existing suppression techniques such as ion doping and the use of solar blind photocathodes (CsTe), the major advantage of using plasmonic filters is that they do not absorb but mainly reflect the target photons, without sacrificing information from the incoming radiation. Thus, a possible architecture could comprise dual-ended readout [44, 45], in which two photodetectors are coupled to opposite sides of the crystal surface. One side of the crystal should implement the plasmonic filter, the other side being as is. Here, the baseline of the filtered side is stabilized, thanks to reflection of the slow component, and can be used as the timing signal. Additionally, the opposite side can be used as the energy signal with a moderate time window when the filtered side triggers a signal.

Considering the effective atomic number and density of BaF_2_, it is insufficient for TOF-PET applications that require a greater density of higher effective atomic number crystals. A recent *metascintillator* innovation could resolve this problem [46–48]. A *metascintillator* comprises stacked dense-slow and light-fast scintillator slabs. Consequently, dense-slow scintillators—the emission spectrum of which does not overlap that of the designed filter—such as bismuth germanate and cerium-doped (Lu,Gd)_3_(Ga,Al)_5_O_12_ (GAGG), could be used to compensate for the relatively low detection efficiency [49, 50].

The angular dependence of the designed filters is a limitation of this study. Scintillation emission is generally isotropic, thus filters should be insensitive to the incident angle. However, making the nano disks insensitive to the incident angle can be challenging. One solution would be to use photonic crystals, directive photonic scintillators having already been proposed and experimentally demonstrated [51–54].

## 3 Conclusions

In this study, we proposed the plasmonic UV filter to suppress the slow component of a BaF_2_ scintillator, which could otherwise degrade the timing performance of BaF_2_-based radiation detectors. We carefully designed the filter by changing the height, period, and diameter of the aluminum nanodisks. The FDTD simulations and experimental results exhibited good agreement. Accordingly, the designed filter can correctly suppress the slow component of the BaF_2_ scintillator. A CTR simulation was conducted from an analytical perspective. The results showed that CTRs better than 100 ps FWHM for all the designed filters could be achieved; indicating that the proposed filters could be used in fast-timing applications. The designed filters not only greatly suppress the slow component but also maintain the usability of it having a lot of information of interacted radiation, maximizing the potential of BaF_2_ scintillator used in fast-timing applications.

## 4 Materials and methods

### 4.1 FDTD simulation

**Figure 6:**
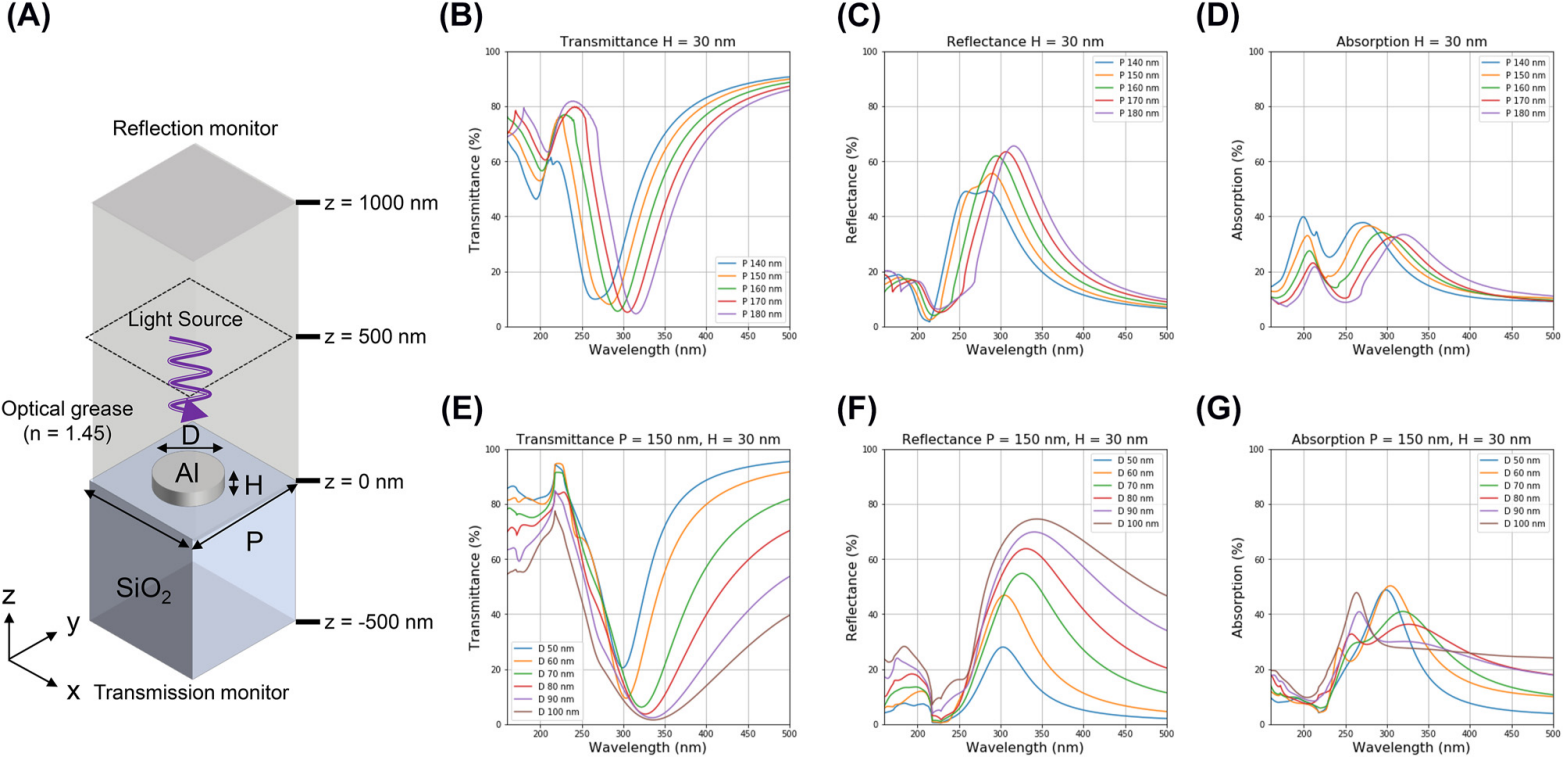
Schematic of FDTD simulation model and simulation results. (A) Schematic of the FDTD simulation for the aluminum nanodisk (B)–(D) FDTD simulation results of the transmittance, reflectance, and absorption spectra of the aluminum nanodisk for periods from 140 to 180 nm (in 10 nm intervals), respectively. (E) and (G) FDTD simulation results of the transmittance, reflectance, and absorption spectra of the aluminum nanodisk for diameters from 50 to 100 nm (in 10 nm steps), respectively.

We conducted FDTD simulations (Ansys Lumerical, Inc., Vancouver) to obtain the best period and diameter specification for the aluminum nano disk. The simulation model is shown in Figure 6(A). The light source is placed at *z* = 500 nm, illuminating the aluminum nanodisk. The reflection and transmission monitors are placed at *z* = 1000 and −1000 nm, respectively. Periodic boundary conditions are set for the *x-, y-*direction, and the perfect matching layer is set for the *z-*direction.

An aluminum nanodisk of height (*H*) 30 nm is placed on a glass substrate and covered with an ideal transparent optical grease considering the practical use of a scintillator. The refractive indices of the aluminum and glass are used in the material data of the simulation, with that of the optical grease being set to 1.45. Figure 6(B)–(D) plots the transmission, reflection, and absorption spectra for the different periods (*P*) of the aluminum nanodisk, from 140 to 180 nm in steps of 10 nm, with the duty ratio (*D/P*, *D* is diameter) being fixed at 0.5. As the period of the nanodisk increases, the reflection peak shifts to longer wavelengths. The relationship between the period and resonance wavelength can be expressed as follows [41]:

(2) $$\lambda_{\text{PSP}}=P\sqrt{\frac{\varepsilon_{m}\left(\lambda \right)\varepsilon_{d}}{\varepsilon_{m}\left(\lambda \right)+\varepsilon_{d}}},$$

where *P* denotes the period of the nanodisk array, and *ε*_m_ and *ε*_d_ denote the permittivity of the metal and dielectric, respectively.

Figure 6(E)–(G) plots the transmission, reflection, and absorption spectra for different diameters (*D*) of the aluminum nanodisks (50–100 nm) with a height (*H*) of 30 nm when the period (*P*) of the nanodisk is fixed to be 150 nm. Effective suppression of the wavelength of the slow component (*λ* = 300 nm) is evident. As the diameter of the nanodisk increases, the reflection peak shifts to longer wavelengths and the FWHM broadens because of the adjacent nanodisk crosstalk [42].

### 4.2 Analytical CTR simulation

**Figure 7:**
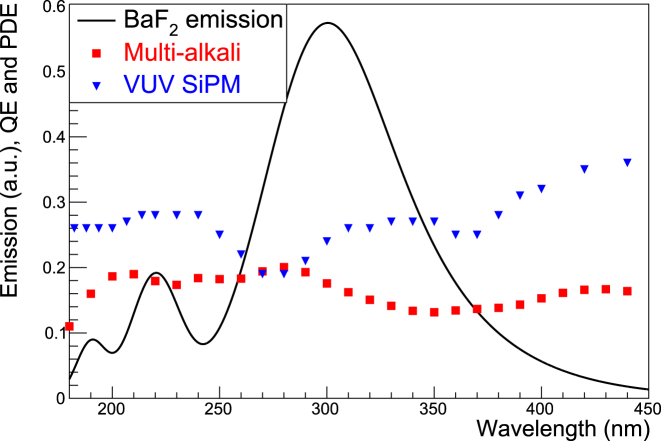
Spectra of BaF_2_ emission, PDE of VUV SiPM, and QE of multi-alkali photocathode modeled in this study. Additionally, an ideal photodetector, the QE of which is 100% over the entire wavelength, is modeled. These spectra were used for the analytical CTR simulations.

The performance of the designed filters was evaluated by multiplying the BaF_2_ emission spectrum, the simulated filter transmission spectra, and the photodetector detection spectra. The BaF_2_ emission spectrum was approximately reproduced according to Ref. [23]. We modeled three types of photodetectors—that is, an ideal photodetector (QE = 100% over the entire wavelength), an MCP-PMT with multi-alkali photocathode, and a VUV SiPM. Figure 7 shows the BaF_2_ emission spectrum and QE/PDE curves used in this study. Note that the multi-alkali QE curve is a typical example rather than a specific manufacturer’s specification.

Additionally, we conducted an analytical CTR simulation of positron-electron annihilation gamma rays to evaluate the designed filters based on the literature discussing analytical CTR [55–57] after calculating the number of filtered photons which could be detected by the abovementioned three types of photodetectors. Mathematical expressions used in the CTR calculations are summarized in the Supplementary Material. However, as important parameters, the SPTR values of the modeled MCP-PMT and VUV SiPM were set to 22 and 70 ps FWHM, respectively, based on Refs. [43, 58], both being state-of-the-art time resolutions. Additionally, the properties of the BaF_2_ scintillator are listed in Table 1. The best CTR (minimum number) was investigated for all combinations of filters and photodetectors (7 filters including *without-filter* (*D* from 50 to 100 nm with a fixed *P* of 150 nm) × 3 modeled photodetectors).

## List of abbreviations

BaF_2_
: Barium fluoride
CTR: coincidence time resolution
EOT: extraordinary optical transmittance
ELT: extraordinarily low transmittance
FWHM: full width at half maximum
FDTD: finite-difference time-domain
LSO: lutetium oxyorthosilicate
LYSO: lutetium–yttrium oxyorthosilicate
MCP: microchannel plate
PDE: photodetection efficiency
PET: positron emission tomography
PMT: photomultiplier tube
QE: quantum efficiency
SiPM: silicon photomultiplier
SEM: scanning electron micrographic
SPP: surfaceplasmon polariton
SPTR: single photon time resolution
TOF: time-of-flight
VUV: vacuum ultraviolet

## Supplementary Material

Supplementary materials include the FDTD simulation of the aluminum nanodisk height-dependent transmittance, aluminum nanodisk fabrication procedure, transmittance measurement, and analytical CTR calculations.

## Supplementary Material

Supplementary Material Details
