# Supplementary material for: Plasmonic ultraviolet filter for fast-timing applications
Source: Nanophotonics. 2023 Jan 23;12(4):743–52. doi: 10.1515/nanoph-2022-0704 (PMC11636206; doi:10.1515/nanoph-2022-0704)
Supplement: Supplementary file 1 — Supplementary Material Details [file j_nanoph-2022-0704_suppl.pdf]

Supplementary material

Plasmonic Ultraviolet Filter for Fast-Timing Applications

Ryosuke Ota*, and Soh Uenoyama

*Corresponding author: Ryosuke Ota, ryosuke.ota@crl.hpk.co.jp
Central Research Laboratory, Hamamatsu Photonics K.K.
5000 Hirakuchi, Hamakita-ku, Hamamatsu City 434-8601, Japan

1 Height-dependent transmittance of the aluminum nano disk

FDTD simulations were conducted to analyze the height-dependent transmittance characterization of the aluminum nano disk while maintaining the diameter (D = 90 nm) and period (P =150 nm) of the aluminum nano disk. **Figure S1(a)** shows the configuration of the FDTD simulation and **S1(b)** shows the simulation results of the aluminum transmission.

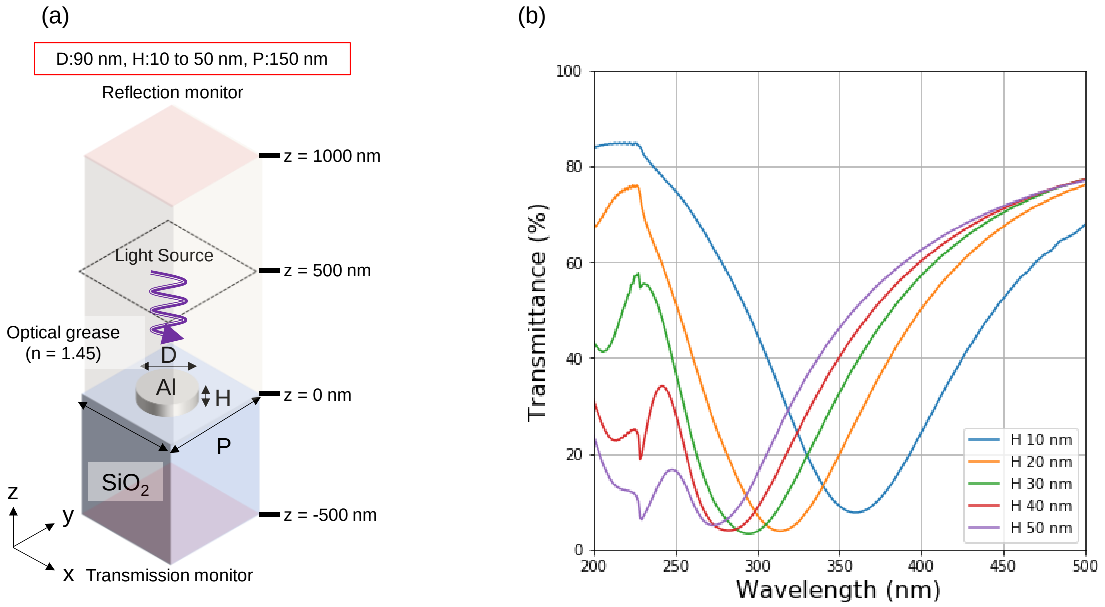

Figure S1. (a) FDTD simulation configuration (b) Height-dependent FDTD transmission simulation.

2 Aluminum nano-disk fabrication procedure

An aluminum nano disk was fabricated using electric-beam (EB) lithography. First, an electron beam resist (ZEP-530A6, ZEON Corps) was coated, the thickness of the EB resist being 140 nm. Next, nano-disk patterning was conducted using an EB irradiation lithography system, the beam current and acceleration voltage being set to 400 pA and 100 keV, respectively. After EB irradiation and development, aluminum was deposited by electron beam deposition, the thickness of the aluminum being 30 nm. Finally, an aluminum nano disk was realized on a glass substrate after the EB resist was removed using the solvent.

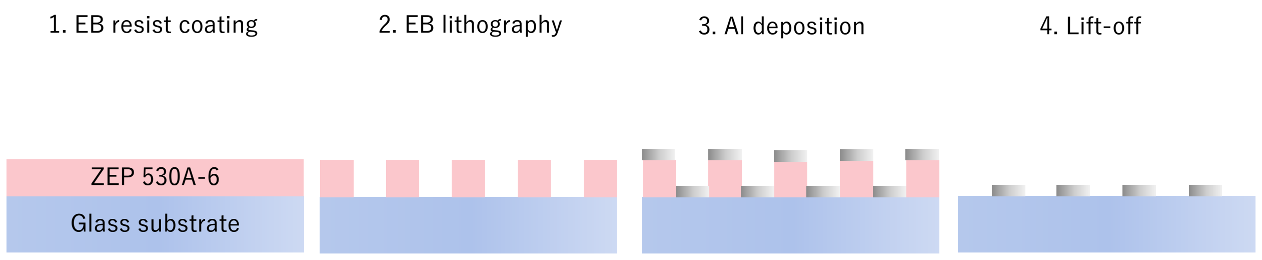

Figure S2. Illustration of the fabrication procedure for the aluminum nano disk.

3 Transmittance measurement

Figure S3 illustrates the experimental setup for measuring the transmittance of the designed filters. The designed plasmonic filters with different diameters of 85, 95, and 105 nm were fabricated on a glass substrate with dimensions of 21.8 mmϕ × 3.0 mm thickness. The glass substrate with the plasmonic filters was mounted on an optical setup of a spectrophotometer (UH4150, Hitachi High-Technologies Corporation). As the fabricated plasmonic filters were too small (900 × 900 μm^2) to directly measure the transmittance of the filters, an aperture with a size of approximately 2.3 × 2.3 mm^2 was inserted between the glass substrate and an integrating sphere. The aperture size was set to the smallest for maximizing the filling factor of the fabricated plasmonic filter. The transmittance measurements were conducted eight times at wavelengths of 200–500 nm and averaged for each plasmonic filter for reducing statistical errors. Finally, the measured data were normalized so that the measured data at 500 nm correspond to the simulation data at 500 nm. This was done due to a difficulty of directly obtaining absolute transmittances.

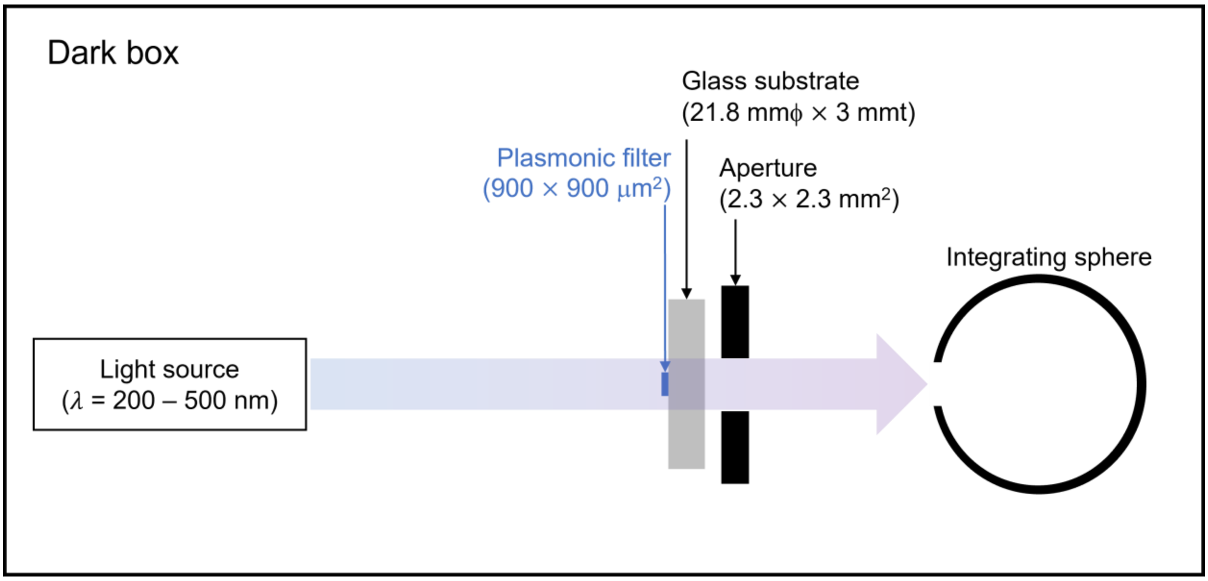

Figure S3. Experimental setup for measuring the transmittance of the designed filters. The sizes are not to scale.

4 Analytical CTR simulation

In this analytical simulation, the kinetics of the BaF2 emission were defined as follows:

$$f(t) = \sum_{i=1}^{2} R_i \frac{e^{-t/\tau_{\mathrm{decay}\_i}} - e^{-t/\tau_{\mathrm{rise}}}}{\tau_{\mathrm{decay}\_i} - \tau_{\mathrm{rise}}},$$

where R_i and τ_{decay_i} denote the light yield and decay time constant of the ith component (i = 1 → fast, i = 2 → slow), respectively, and τ_{rise} denotes the rise time constant.

The emission wavelengths of the two fast and one slow components were set to be approximately 195/220 and 300 nm, respectively. The parameters used in the simulations are listed in **Table 1**. Note that in the case of the modeled photodetectors, R_i was weighted according to the QE/PDE curves depicted in **Figure 3**. Assuming that the temporal response of photodetectors to a single photon forms a Gaussian distribution, the temporal response *r(t)* can be expressed as follows:

$$r(t) = \frac{1}{\sqrt{2\pi}\sigma} e^{-\frac{1}{2}\left(\frac{t-\bar{t}}{\sigma}\right)^2},$$

where $\bar{t}$ denotes the expected time from detection to readout and σ denotes the single-photon time resolution (SPTR).

Therefore, the temporal kinetics *f'(t),* including scintillation emission and photodetector detection, can be expressed as follows:

$$f'(t) = (f * r)(t),$$

where *denotes the convolution operator.

In this study, the SPTRs for an MCP-PMT and a VUV SiPM were set to 22 and 70 ps FWHM, respectively, based on state-of-the-art detectors. The probability (*P*) that *N* photons are emitted within time (*t*) can be expressed as follows:

$$P_N(t) = \frac{\left(\int_0^t f'(t')dt'\right)^N e^{-\int_0^t f'(t')dt'}}{N!}.$$

Consequently, the probability (*W*) that the Qth photon is emitted within (*t*–*t*+*dt*) can be expressed as follows:

$$W_Q(t)dt = P_{Q-1}(t) \times f'(t)dt$$

Finally, we calculated the estimated coincidence time distribution as follows:

$$P_{\mathrm{Coincidence}}(t) = \int_{-\infty}^{\infty} W_Q(t')W_Q(t+t')dt'.$$

This probability density function can be used as the CTR estimator by fitting a single Gaussian function and extracting the standard deviation or the FWHM. To determine the achievable CTR, *Q* was stepped (in steps of 1), and the minimum CTR was examined for all combinations of filters and photodetectors (seven filters including *without-filter* (*D* from 50–100 nm with a fixed *P* of 150 nm) × 3 modeled photodetectors).
